# Attitudes and practices regarding volunteering among medical graduates from Romania

**DOI:** 10.1186/s12909-026-09234-3

**Published:** 2026-08-20

**Authors:** Andreea Iulia Pop, Lucia Maria Lotrean

**Affiliations:** Department of Community Medicine, Research Center in Preventive Medicine, Health Promotion and Sustainable Development, Iuliu Hatieganu University of Medicine and Pharmacy, Cluj- Napoca, 400012 Romania

**Keywords:** Volunteering, Community health, Medical graduates, Medical school

## Abstract

**Introduction:**

The public health sector is among the most in need of volunteer engagement. The medical student community may participate in many voluntary activities, such as getting involved in public health programs, organizing community health awareness events, delivering administrative support to local health authorities, or engaging in emergencies or in disaster relief operations. Prior studies demonstrated that students’ involvement in extracurricular activities such as volunteering and community service projects can lower burnout, develop stress management skills, and promote leadership, teaching, and soft skills like communication, decision-making, social skills, and empathy; also, volunteering positively impacts academic performance.

**Objectives:**

This study focuses on the assessment of perspectives on the importance of volunteering and factors that influence student participation in voluntary activities as well as on identifying volunteering related behaviors among medical graduating students. The study aims to compare behaviors and attitudes related to involvement in voluntary activities between Romanian and international graduates and analyze these factors throughout time from 2021 to 2023.

**Methods:**

A cross-sectional study was conducted between 2021 and 2023 among 572 medical graduates at the Iuliu Hațieganu University of Medicine and Pharmacy (IHUMP) in Cluj-Napoca, Romania.

**Results:**

The outcomes demonstrate that most graduates recognized the significance of volunteering. Factors promoting engagement in volunteering activities, as more strongly recognized by students, include valuing personal motivation, appreciating socializing opportunities, and recognizing the desire to offer community assistance. Over 70% of graduates reported volunteering, with significantly higher participation among Romanian graduates (> 80%) compared to international graduates (< 50%). Half of the graduates were interested in participating in voluntary activities after graduation, international graduates being statistically significant more interested in this. Romanian students were more engaged in student exchanges, student organizational and administrative activities, health education and medical support initiatives, as well as volunteering in emergency and medical assistance services, while international students were more inclined towards social and humanitarian volunteering activities.

**Conclusion:**

This study indicates that enhancing educational and cultural strategies could improve voluntary participation among medical students. This study offers significant insights for mentors, policymakers, and graduates, promoting a collaborative approach to improve medical education and volunteer participation.

**Supplementary Information:**

The online version contains supplementary material available at 10.1186/s12909-026-09234-3.

## Introduction

Volunteering is characterized as a deliberate action undertaken without professional obligation, aimed at supporting, preserving, and promoting social values, without expectation of rewards [1]. Youth participation in volunteering has become increasingly significant across multiple domains. A more comprehensive definition of volunteerism refers to: “voluntary action, little to no compensation, longevity, planfulness, non-obligation, and organizational context’’ [2].

One of the neediest sectors for volunteer involvement is by far public and community health [3]. Medical students may contribute by getting involved in public health programs, organizing awareness events, assisting local health authorities, or engaging in disaster relief operations [4]. Furthermore, student volunteering serves as an effective strategy for managing health crises, such as pandemics, when limited system capacity made student engagement essential [5, 6].

Decades of study have been conducted on volunteering and community participation from a psychological standpoint. The prevalent agreement is that the altruistic efforts of volunteers provide several advantages to both communities and the volunteers themselves [2, 7]. Volunteering is associated with lower burnout, improved stress management, leadership, teaching, and soft skills like communication and decision-making, as well as better academic performance [4, 8]. It also enhances professional development according to Kolb’s experiential learning theory which highlight that real-life situations helps to consolidate knowledge and competencies [9, 10].

Medical students worldwide engage in various structured volunteer initiatives, including those organized by the International Federation of Medical Students’ Associations, the Street Medicine Institute, student-run free clinics, Global Brigades, Médecins Sans Frontières, Red Cross and Red Crescent Student Chapters, and the Medical Reserve Corps in the USA [11]. These are a few examples of international organizations that provide medical students with diverse volunteer options, ranging from free student-run clinics to international public health.

In Romania, medical students may participate in voluntary activities, including student exchanges, organizational and administrative tasks, health education and medical support initiatives, and assisting in emergency and medical assistance services.

We performed an investigation prior to the pandemic period, in 2018, among third- and fifth-year medical students from the Romanian section of Iuliu Hatieganu University of Medicine and Pharmacy (IHUMP), which revealed that over 80% of the former group and more than two-thirds of the latter group participated in various voluntary activities during their medical education [12]. Afterwards, another study was performed between June and July 2021 on the involvement of medical students from IHUMP in voluntary activities during the COVID pandemic and showed that 20% of medical students participated in voluntary activities during the pandemic. They participated in hospital activities, worked in the public health directorate, and assisted in patient follow-up in collaboration with general practitioners. Volunteering students carried out health education activities, assisted in delivering medicines and food to isolated or quarantined people, and worked in vaccination institutions [13].

Nevertheless, medical students preferred voluntary activities, their level of participation during medical school, and their willingness to continue volunteering after graduation still need to be assessed. This study, focusing on graduates, serves as a balance sheet to evaluate their full volunteer involvement.

This investigation will help to ascertain if and how medical education should incorporate voluntary work, within the framework in which some organizations saw students as having flexible schedules, youthful enthusiasm, and a skill level appropriate to their academic pursuits, and they are ready to be involved in voluntary activities [14].

A previous study of Irish medical students’ willingness to volunteer found that they were more likely to do so if they felt altruistic (cared about other people’s well-being or wanted to help those in need), professional development (wanted to learn new skills, get training in emergency response, and work in a new setting), career (wanted to improve their CV, increase their job prospects, and make connections), community social duty (lessened feelings of guilt over helping the less fortunate), and improvement (more confidence in similar situations) [3]. To our knowledge, no research in the literature addresses the perspectives of Romanian medical graduates towards participation in voluntary activities. Knowledge of student interest and attitudes toward volunteering can aid policymakers and non-governmental organizations in effectively developing healthcare volunteer teams involving medical students.

The current study aims to investigate attitudes and practices related to medical students’ engagement in voluntary activities. It also looks at possible differences between Romanian and international graduates in three graduating cohorts (2021–2023). This study, while not the first of its kind in Europe, represents the inaugural investigation in Romania concerning the attitudes and behaviors of medical graduates engaged in voluntary activities during their medical education until graduation. The present study was designed to use a survey applying 5-point Likert scales and multiple-choice questions to assess the attitudes influencing voluntary engagement and behaviors among graduates from 2021 to 2023, while also examining their socio-demographic variables.

This research conducted a targeted assessment of the subsequent objectives:

- Assessment of attitudes about the importance of volunteering and factors that encourage medical graduates’ participation in voluntary activities: personal, community, and educational influences.
- Identifying volunteering related behaviors: participation in voluntary activities during medical school, type of voluntary activity, and interest in participating in voluntary activities after graduation.
- Comparing behaviors and attitudes for involvement in voluntary activities between Romanian and international graduates and analyze these factors throughout time from 2021 to 2023.

## Methods

### Sample and procedure for data collection

The current study is cross-sectional investigation and constitutes a part of broader research designed to evaluate medical graduates’ involvement in research and voluntary activities. The project was ethically approved by the Iuliu Hatieganu University of Medicine and Pharmacy Ethics Commission under Approval Number DEP27/03.11.2021.

The university’s Faculty of Medicine accepts three cohorts per year and offers Romanian, English, and French sections. Student selection varies per section. The Romanian section admits students by a standardized examination. International English and French language candidates are accepted based on academic performance and personal achievements [15].

The current investigation centers on the medical graduates from both the Romanian and international sections of the University of Medicine and Pharmacy, Iuliu Hațieganu, in Cluj-Napoca, Romania. The eligibility criteria were medical graduates enrolled in Romanian and international sections of Faculty of Medicine who finished their studies in 2021, 2022 and 2023. Data was collected by an anonymous online questionnaire via the Teams platform, commonly utilized by members affiliated with the University of Medicine and Pharmacy, Iuliu Hațieganu. The questionnaire was administered for each cohort at the end of the last university year. All graduates from the Faculty of Medicine (Romania, English and French section) were invited to participate. We informed the graduates that their participation was optional, and we requested their consent to participate through the completion of the questionnaire. Individuals who opted out of participation did not complete the questionnaire. Graduates received the questionnaire in Romanian, English, and French, and the average time to complete it was 15–20 min.

We calculated the minimum number of participants needed and obtained statistically valid findings using the free online program Open Epi Version 3.01 [16]. For this survey, we invited 1878 graduates overall from the target population. The study needs a minimum of 491 participants with a 99% confidence level and a ± 5% margin of error. A total of 572 graduates represents the final sample for this study. This cuts the risk of sample bias, therefore improving the generalizability of the results, and shows their reliability.

### Instrument for data collection

This research project involved the development of an online survey to assess socio-demographic variables (age, gender), academic variables (section, year of graduation), perceptions on attitudes that encourage or discourage volunteer engagement, and the volunteer activities of medical graduates. We performed a comprehensive literature analysis to identify common topics and characteristics reported in prior studies, enabling us to identify the motivational attitudes associated with medical graduates’ participation in volunteering. This influenced the design and creation of our survey items (see Supplementary File 1).

The study investigated opinion of participants about and the importance of volunteering as well as attitudes regarding the factors that encourage medical graduates involvement in volunteering, the factors included in the questionnaire being the following: personal influence (motivation and personal interest, curriculum vitae improvement motivation, desire to improve clinical knowledge and skills, desire to gain experience in clinical reasoning and case management), community influences (example of other colleagues, the desire to help the community), educational influences (teacher presentation of volunteering participation options, desire to contribute to the medical education of different population groups). The response choices regarding the importance of volunteering were presented on a four-point scale that varied from “very important” to “not at all important” (very important, coded + 2, important, coded + 1, undecided, coded 0, not at all important, coded − 1). The response choices regarding the factors were presented on a five-point scale that varied from “strongly disagree” to “strongly agree” (strongly agree, coded + 2, agree, coded + 1, undecided, coded 0, disagree, coded − 1, strongly disagree, coded − 2).

The questionnaire included also the voluntary practices of medical graduates. We asked the graduates if they had engaged in voluntary activities during their medical school years. The questionnaire aimed to gather data about types of organizations and types of activities (student exchanges, student organizational and administrative activities, health education and medical support activities, volunteering in the emergency and medical assistance service, social-humanitarian activities or non-medical activities). The response choices were ‘Yes’ or ‘No’ (Yes-coded 1, No-coded 0). The questionnaire also included one open-ended item, where graduates could specify the types of organizations or activities they were involved in. Some graduates provided full explanations of the initiatives they mentioned, while others only indicated the name or acronym. To ensure clarity, we organized a table which includes the complete name of each initiative, followed in parentheses by a short description. These descriptions are based either on the information written directly by the participants or, when initiatives were only named/acronymized, on supplementary data collected from official sources (websites, project pages, organizational materials) in order to adequately describe them. For certain initiatives where no further information could be found and participants did not provide explanations, the content was marked as “Not specified”.

Furthermore, the questionnaire assessed the motivation and interest to participate in voluntary activities after completing medical studies (with response options being ‘Yes’-coded 1, No’- coded − 1 or ‘I do not know’-coded 0) (see Supplementary File 1).

We assessed how reliable the questionnaire was by analyzing its internal consistency and finding Cronbach’s alpha for both the practice variables and the variables about attitudes toward volunteering. We found that the perspectives on volunteering and the behavior, which included 14 items, had a Cronbach’s alpha of 0.71, which indicates good internal consistency. Our prior research, among medical students in their third and fifth years of study, tested for the first time the present questionnaire. We made small modifications to coincide with the actual study topics, improving the questionnaire’s clarity and reliability.

### Data analyses

Prevalence and mean values were determined distinctly for both sections, as well as for graduates from the 2021, 2022, and 2023 cohorts. Chi^2^- tests and T-tests were employed to examine disparities among graduates in the Romanian and International sections, as well as among graduates from the 2021, 2022, and 2023 cohorts.

Two multivariate linear regression analyses were performed to determine the attitudes associated with involvement in voluntary activities during medical school and to analyze the attitudes associated with involvement in voluntary activities after graduation. The dependent variables were volunteering involvement during medical school and interest in involvement in voluntary activities after graduation.

For both multivariate linear regression analyses, the independent variables were: socio-demographic variables (age, gender - coded 1 for males and 2 for females), academic variables (section - Romanian section- coded 1 and international section- coded 2, year of graduation- 2021, 2022, 2023), opinion about the importance of volunteering, attitudes related to personal influences (motivation and personal interest, curriculum vitae improvement motivation, desire to improve clinical knowledge and skills, desire to gain experience in clinical reasoning and case management.), community influences (example of other colleagues, the desire to help the community), and educational influences (teacher presentation of volunteering participation options, desire to contribute to the medical education of different population groups).The analyses were performed separately for each linear regression analysis and stepwise forward selection was used.

The data was analyzed utilizing SPSS 26 statistical software, with significant findings reported at a significance level of 0.05.

## Results

The total sample included 572 graduates, reflecting a response rate of around 30% from the invited sample. Among the participants, 392 (68.5%) were graduates from the Romanian section, while 180 (31.5%) were from the international section; 232 (40.5%) students graduated in 2021, 172 (30%) in 2022, and 168 (29.5%) in 2023; 215 (37.6%) were male and 357 (62.4%) were female, with ages ranging from 22 to 54 years (mean 25.25, SD 2.1).

### Attitudes towards involvement in voluntary activities

More than 90% of graduates rated volunteering as important or very important. Over 70% of graduates strongly agreed that volunteering is primarily driven by personal motivation and interest. Between 33.9% and 40% of graduates strongly agreed that curriculum vitae improvement motivates students to volunteer. Between 42.9% and 52.2% of graduates strongly agreed that students perceive volunteering as an opportunity to improve their clinical knowledge and skills and to gain experience in clinical reasoning and case management. Approximately 40% strongly agreed that the example of other colleagues who volunteer is important. More than half strongly agreed that volunteering is valued for the opportunity to socialize, to build interpersonal relationships, and to help the community. Approximately a quarter of the respondents strongly agreed that the teaching staff provided opportunities to volunteer. Finally, between 41.3% and 51.3% strongly agreed that contributing to medical education of different population groups was an influential motivation.

The comparative analysis indicated statistically significant differences between the Romanian and international sections: international graduates were more convinced about the role played by the desire to gain experience in clinical reasoning and case management, teacher presentation of volunteering participation options, and a desire to help the community, whereas Romanians recognized more the influences from other colleagues. A statistically significant difference exists between the cohorts of 2021 and 2022 graduates, 2021 graduates showing a greater influence from the desire to contribute to the medical education of different population groups (See Table 1).

**Table 1:** Attitudes about the importance of volunteering and influences supporting medical graduates’ involvement in volunteering

	2021	2022	2023	Romanian graduates	International graduates
Importance of volunteering (%)					
Very important (%) ^i^	50.4	55.2	45.8	53.1	45.0
Important (%) ^ii^	42.2	38.4	48.2	40.8	47.2
Undecided (%) ^iii^	5.6	3.5	2.4	3.1	6.1
Not at all important (%) ^iv^	1.7	2.9	3.6	3.1	1.7
Mean	1.41	1.46	1.36	1.44	1.36
Factors which influence students envolvement in voluntering					
Personal influence					
Motivation and personal interest (%)					
Strongly agree (%) ^i^	76.3	78.5	72.6	75.8	76.1
Agree (%) ^ii^	21.1	18.0	25.6	21.4	21.7
Undecided (%) ^iii^	1.3	0.6	0.6	0.5	1.7
Disagree (%) ^iv^	1.3	2.9	1.2	2.3	0.6
Strongly disagree -	0	0	0	0	0
Mean	1.72	1.72	1.70	1.71	1.73
Desire for improving CV (%)					
Strongly agree (%) ^i^	40.9	34.9	33.9	39.3	32.2
Agree (%) ^ii^	35.8	42.4	42.3	39.3	40.6
Undecided (%) ^iii^	5.6	5.2	2.4	3.3	7.2
Disagree (%) ^iv^	17.7	17.4	21.4	18.1	20.0
Strongly disagree -	0	0	0	0	0
Mean	1.00	0.95	0.89	1.00	0.85
Desire to improve clinical knowledge and skills (%)					
Strongly agree (%) ^i^	52.2	46.5	42.9	45.2	53.3
Agree (%) ^ii^	33.2	35.5	38.7	37.8	30.6
Undecided (%) ^iii^	4.7	1.7	4.2	2.8	5.6
Disagree (%) ^iv^	9.9	16.3	14.3	14.3	10.6
Strongly disagree -	0	0	0	0	0
Mean	1.28	1.12	1.10	1.14	1.27
Desire to gain experience in clinical reasoning and case management (%)					
Strongly agree (%) ^i^	51.7	43.6	44.6	43.6	55.0
Agree (%) ^ii^	31.5	39.0	37.5	37.2	31.7
Undecided (%) ^iii^	5.2	2.3	4.2	3.8	4.4
Disagree (%) ^iv^	11.6	15.1	13.7	15.3	8.9
Strongly disagree -	0	0	0	0	0
Mean	1.23	1.11	1.13	1.09^d^	1.33
Community influences					
Influences from other colleagues(%)					
Strongly agree (%) ^i^	47.4	39.5	44.0	48.5	34.4
Agree (%) ^ii^	36.2	47.1	41.1	37.8	47.8
Undecided (%) ^iii^	2.2	1.7	2.4	0.8	5.0
Disagree (%) ^iv^	14.2	11.6	12.5	13.0	12.8
Strongly disagree -	0	0	0	0	0
Mean	1.17	1.15	1.17	1.22^d^	1.04
The opportunity for socialization and interpersonal relationships (%)					
Strongly agree (%) ^i^	56.9	53.5	54.8	57.7	50.0
Agree (%) ^ii^	34.5	42.4	39.3	36.5	42.2
Undecided (%) ^iii^	1.3	1.2	0.6	0.5	2.2
Disagree (%) ^iv^	7.3	2.9	5.4	5.4	5.6
Strongly disagree -	0	0	0	0	0
Mean	1.41	1.47	1.43	1.46	1.37
The desire to help the community (%)					
Strongly agree (%) ^i^	56.5	52.3	51.2	50.0	61.7
Agree (%) ^ii^	32.3	33.7	37.5	37.5	27.2
Undecided (%) ^iii^	4.3	2.9	2.4	2.0	6.1
Disagree (%) ^iv^	6.9	11.0	8.9	10.5	5.0
Strongly disagree -	0	0	0	0	0
Mean	1.38	1.27	1.31	1.27^d^	1.46
Educational influences					
Teacher presentation of volunteering participation options (%)					
Strongly agree (%) ^i^	25.4	21.5	21.4	19.1	31.7
Agree (%) ^ii^	39.2	37.2	36.3	35.7	42.2
Undecided (%) ^iii^	3.0	2.9	5.4	1.3	8.9
Disagree (%) ^iv^	32.3	38.4	36.9	43.9	17.2
Strongly disagree -	0	0	0	0	0
Mean	0.58	0.42	0.42	0.30^d^	0.88
Desire to contribute to the medical education of different population groups (%)					
Strongly agree (%) ^i^	51.3	41.3	46.4	44.6	51.7
Agree (%) ^ii^	36.6	41.9	42.3	42.6	33.9
Undecided (%) ^iii^	4.7	3.5	3.6	2.8	6.7
Disagree (%) ^iv^	7.3	13.4	7.7	9.9	7.8
Strongly disagree -	0	0	0	0	0
Mean	1.32^a^	1.11	1.27	1.22	1.29

Legend: i coded + 2, ii coded + 1, iii coded 0, iv coded -1

a = *p* < 0.05—statistically significant differences at t-test between 2021–2022 graduates

d= *p* < 0.05—statistically significant differences at t-test between Romanian and International graduates

### Practices related to graduates’ involvement in voluntary activities

More than 70% of graduates were involved in voluntary activities during medical school, with Romanian graduates participating at a rate over 80%, comparing with international graduates which were engaged at a rate below 50%. The majority chose participation in voluntary activities within the faculty’s medical student organization, with a statistically significant difference favoring Romanian graduates. Over half of the graduates were interested in participating in voluntary activities after graduation, international graduates being statistically significant more interested in this (See Table 2). 

**Table 2:** Graduates’ involvement in voluntary activities during medical school and intentions after graduation

	2021		2022	2023	Romanian graduates	International graduates
Volunteering involvement (%)	72		76.7	71.4	85.2e	47.2
Involvement in voluntary activities during medical school within Medical Students’ Organization from Cluj (%)	54.7		63.4	61.3	78.1^e^	18.3
Involvement in voluntary activities during medical school within another organization (%)	41.4	44.8		38.1	42.6	38.9
Interested in participating in voluntary activities after graduation						
Yes (%)	53.9	56.4		56.5	50.0	67.2
I don’t know (%)	30.6	25.6		21.4	34.4	8.9
No (%)	15.5	18.0		22.0	15.6	23.9
Mean	0.38	0.38		0.35	0.34^d^	0.43

Legend: d = *p*<0.05- statistically significant differences at t-test betweeen Romanian and International graduates; e = *p*<0.05- statistically significant differences at chi^2^-test between Romanian and International graduates

Regarding the activities participated in by graduates, most Romanian graduates from all cohorts preferred student exchanges, student organizational and administrative activities, health education and medical support initiatives, and volunteering for emergency and medical assistance services, while international graduates preferred social-humanitarian activities. (See Table 3).

**Table 3:** Type of voluntary activities

	2021	2022	2023	Romanian graduates	International graduates
Student exchanges, student organizational activities and administrative activities (%)	30.1	30.8	29.2	29.9^e^	11.1
Health education and medical support activities (%)	19.8^b^	24.4^c^	26.8	29.9^e^	8.9
Volunteering in the emergency and medical assistance service (%)	24.5	27.4^c^	14.3	30.3^e^	5
Social-humanitarian activities (%)	11.2^a,b^	5.8^c^	8.9	5.9^e^	15.5
Non-medical (%)	6.9	6.9	7.1	7.7	5.6

Legend:  a= *p* < 0.05—statistically significant differences at chi^2^-test between 2021–2022 graduates ; b = *p* < 0.05—statistically significant differences at chi^2^-test between 2021–2023 graduates ;  c= *p* < 0.05—statistically significant differences at chi^2^-test between 2022–2023 graduates ;  e= *p* < 0.05—statistically significant differences at chi^2^-test between Romanian and International graduates

Table 4 provides a summary of the key activities, projects, and associations highlighted by graduates as part of their volunteering and student support experiences.

**Table 4:** Overview of Volunteering activities and support for students

Category		Activity		Projects	
1.Student exchanges, student organizational activities and administrative activities	*N* = 181 (32.3%)	Administration and coordination	*N* = 56 (9.5%)	• Medical Students’ Organization from Cluj (Representative student organization of the Faculty of Medicine in Cluj, promoting education, scientific, mobility, and community projects)	*N* = 45 (7.9%)
				• The International Federation of Medical Students’ Associations (international organization that advocates for medical students, concentrating on education, public health, and cultural exchange)	*N* = 1 (0.2%)
				• Federation of Medical Students’ Associations in Romania (national organization with a focus on promoting education, health, and advocacy)	*N* = 3 (0.5%)
				• European Medical Students´ Association (European network of medical students, promoting medical education, health policy, ethics, and mobility)	*N* = 11 (1.9%)
		Involvement in organizing conferences	*N* = 59 (10.3%)	• Medicalis Conference (international congress designed for students and young health professionals)	*N* = 23 (4%)
				• Medical Education Days (medical conferences and workshops)	*N* = 14 (2.4%)
				• Healthcare, Education and Research Talks Conference (educational and mentoring initiative for students and young doctors, combining research, education, and clinical practice)	*N* = 27 (4.7%)
		Workshops and Seminars organizing	*N* = 53 (9.2%)	• The Romanian Surgical Student Society (developing practical surgical training methods for students)	*N* = 19 (3.3%)
				• Organizing scientific summer camps for foreign students – Not specified	*N* = 2 (0.4%)
				• Medical Hackathon Cluj (is uniting students, IT experts, engineers, entrepreneurs, and designers to create groundbreaking healthcare solutions)	*N* = 2 (0.4%)
				• Medical Research Education (emphasizes the enhancement of research abilities, methodologies, and scientific communication skills)	*N* = 29 (5.1%)
				• Students’ Association of Robotic Urologic Surgery (engages students, residents, and professionals through workshops, conferences, and mentorship in robotic/urologic and advanced surgical techniques)	*N* = 5 (0.9%)
				• Breaking the silence’ (initiative teaching Romanian Sign Language to foster inclusion of the deaf community)	*N* = 2 (0.4%)
		Scientific Circles attendance and organizing	*N* = 4 (0.7%)	• Scientific circle of anatomy	*N* = 1 (0.2%)
				• Scientific circle of physiology	*N* = 1 (0.2%)
				• Scientific circle of infectious diseases	*N* = 1 (0.2%)
				• Scientific circle of bioethics	*N* = 2 (0.3%)
		Student exchanges and guidance	*N* = 63 (10.6%)	• ‘Tutors for the New Generation’ (aims to make the transition smoother for new students by providing them with guidance and support from more experienced peers.)	*N* = 10 (1.8%)
				• Medical Corporation of Cluj (Francophone student association fostering solidarity, integration, and social life among French-speaking healthcare students)	*N* = 13 (2.3%)
				• ‘Living Fire’ (social event project)	*N* = 13 (2.3%)
				• TransMed (student exchanges between medical schools in Romania)	*N* = 16 (2.8%)
				• The International Student Exchanges Project	*N* = 18 (3.1%)
				• Twinning Project (intercultural exchange of experience)	*N* = 4 (0.7%)
				• Standing Committee on Research Exchange) (International exchange program offering research internships for medical students)	*N* = 1 (0.2%)
				• Standing Committee on Professional Exchange (international clinical exchange opportunities for medical students.)	*N* = 2 (0.3%)
2. Health education and medical support activities	*N* = 116 (20.3%)	Medical education in schools and kindergartens	*N* = 109 (18.4)	• ‘The Big Ones for The Little Ones’ (UMPIH initiative supporting high school students from disadvantaged rural backgrounds with workshops, lab visits, mentoring, cultural activities, and free accommodation)	*N* = 3 (0.5%)
				• ‘Teddy bear hospital’ (project for preschoolers, reducing fear of doctors and hospitals through fun pretend-play activities where children act out medical roles)	*N* = 16 (2.8%)
				• “Little Health Workers’ (a project designed for primary school children, focusing on health awareness through enjoyable educational activities with a medical theme)	*N* = 54 (9.4%)
				• “Little people (Psychosocial support for pediatric oncology patients—daily activities, volunteer-run programs, hospital playroom renovations, family support, and survivor clubs)	*N* = 2 (0.3%)
				• Junior Summer MedSchool (UMPIH initiative summer camp for high school students simulating medical student life)	*N* = 4 (0.7%)
				• 3 S Project (educational campaign on sexual health and prevention through open and informed discussions)	*N* = 5 (0.9%)
				• Other educational activities (Health education project in schools and high schools, promoting prevention and healthy lifestyles) – Not specified	*N* = 40 (7%)
		Public health, information campaigns	*N* = 84 (14.1%)	• ‘Donate Blood! Be a Hero!’ Project (initiative promoting blood donation nationwide through awareness campaigns and donation drives at transfusion center)	*N* = 70 (12.2%)
				• ‘Road safety’ (educational campaign targeting the public to promote road safety awareness and safe behaviors)	*N* = 20 (3.5%)
				• Vaccination marathon (vaccination campaign during the COVID 19 pandemic)	*N* = 5 (0.9%)
		Basic life support courses	*N* = 9 (1.5%)	• The Resuscitation Marathon (first-aid training sessions taught by volunteer medics and paramedics)	*N* = 9 (1.5%)
3.Volunteering in the emergency and medical assistance service	*N* = 128 (21.6%)	Screening programs in vulnerable communities	*N* = 65 (10.9%)	• ‘Together for Health’ (project aimed at supporting basic medical education by providing accessible health resources to the broader community)	*N* = 22 (3.9%)
				• ‘Doctors for You’ (medical students and professionals work together to deliver free public lectures about preventing and treating illnesses)	*N* = 26 (4.6%)
				• The Caravan with Doctors Association (mobile medical clinic providing free primary and specialized healthcare, screenings, and health education to underserved rural communities)	*N* = 26 (4.6%)
				• Omnis foundation (non-profit providing medical aid and educational support in crisis-stricken communities)	*N* = 3 (0.5%)
		Medical care in vulnerable environments	*N* = 17 (2.9%)	• Medical assistance (healthcare in hospitals or in Public Health Directorate of Cluj County during the pandemic and screening campaigns)	*N* = 15 (2.6%)
				• Oncohelp (Student volunteers support fundraising activities to sustain oncology services and patient care)	*N* = 2 (0.4%)
		Emergency rescue service	*N* = 56 (8.8%)	• Mobile Emergency Service for Reanimation and Extrication	*N* = 25 (4.4%)
				• Local Ambulance Service	*N* = 35 (6.1%)
				Disaster medicine (focused on educating the wider public about disaster medicine principles and emergency preparedness)	*N* = 5 (0.9%)
				• Community Emergency Response Team (program training civilians in disaster preparedness and basic response skills to support professional responders)	*N* = 1 (0.2%)
4.Social-humanitarian activities	*N* = 51 (8.6%)	Social support, health, solidarity, community.	*N* = 51 (8.6%)	• Panda Project organizing fundraising events, donations, and community support)	*N* = 2 (0.3%)
				• Magic Camp Association (organizing summer camp for children with cancer and other serious illnesses, offering recovery, play, and peer support)	*N* = 2 (0.3%)
				• Sport and autism – Health and Fun with 3 C Therapy (Awareness–Coordination–Concentration) – psychomotor therapy for children with autism, improving focus and coordination through simple exercises)	*N* = 2 (0.4%)
				• Red Cross Maltez - Order of Malta (Humanitarian organizations providing emergency aid, health education, and community support through volunteering and relief programs)	*N* = 13 (2.3%)
				• Association of Christian Physicians in Romania (unites doctors, residents, and students for medical missions, conferences, and peer support)	*N* = 1 (0.2%)
				• Other social (fundraising and charity activities) - Not specified	*N* = 33 (5.8%)
5. Non-medical	*N* = 40 (7%)	Non-medical activities	*N* = 40 (7%)	• Sanity in dance steps (Community project using dance to improve seniors’ physical and mental health)	*N* = 7 (1.2%)
				• Other non-medical (Ecological projects, animal welfare actions, social initiatives, and community support)- Not specified	*N* = 40 (6.7%)

* “Not specified” indicates that graduates did not provide the name of the volunteering organization and mentioned only the type of activity

### Factors associated with medical graduates’ volunteer involvement and future interest

The multivariate regression analysis of the 2021 graduate cohort indicates that involvement in voluntary activities was higher among Romanians and among graduates who expressed stronger agreement with the importance of volunteering and with intrinsic motivation and interest.

The analysis of the 2022 graduate cohort indicates that involvement was higher among Romanian graduates and among those who expressed stronger agreement with the importance of volunteering, and among those who expressed stronger agreement with the role of socialization and interpersonal relationships.

Also, the analysis of the 2022 gratuate cohort shows that involvement in voluntary activities was positively associated with lower agreement with the role of volunteering for gaining clinical experience.

Analysis of the 2023 graduate cohort indicates a higher level of involvement in voluntary activities among women and Romanian graduates.

The analysis of the Romanian graduate cohort indicates that involvement was higher among those who expressed stronger agreement with the importance of volunteering and the influence of colleagues. Age was negatively associated with volunteering involvement.

The analysis of the international graduate cohort indicates that involvement was higher among those who expressed stronger agreement with the importance of volunteering and with intrinsic motivation and interest. In this cohort as well, involvement in voluntary activities was positively associated with lower agreement with the role of volunteering for gaining clinical experience (See Table 5).

**Table 5:** Multivariate linear regression - Factors associated with involvement in voluntary activities

Cohort		Beta	CI	*P*
2021 Graduates	Section	-0.409	(-0.516 - -0.302)	< 0.001
	Importance of volunteering	0.111	(0.034 - 0.187)	< 0.001
	Motivation and personal interest	0.109	(0.015 - 0.203)	< 0.01
2022 Graduates	Section	-0.202	(-0.332- -0.072)	< 0.01
	Importance of volunteering	0.196	(0.112 – 0.280)	< 0.05
	Desire to gain experience in clinical reasoning and case management	-0.073	(-0.128 - -0.018)	< 0.05
	The opportunity for socialization and interpersonal relationships	0.111	(0.04–0.199)	< 0.05
2023 Graduates	Section	-0.405	(-0.536 - -0.273)	< 0.001
	Gender	0.191	(0.065 - 0.316)	< 0.01
Romanian Graduates	Importance of volunteering	0.094	(0.045 - 0.142)	< 0.001
	Age	-0.048	(-0.073 - -0.022)	< 0.001
	Influences from other colleagues	0.040	(0.005 - 0.075)	< 0.05
International graduates	Importance of volunteering	0.213	(0.110 - 0.316)	< 0.001
	Desire to gain experience in clinical reasoning and case management	-0.122	(-0.198- -0.046)	< 0.01
	Motivation and personal interest	0.208	(0.070 - 0.346)	< 0.01

Legend: Statistical significance was considered at *p* < 0.05

The multivariate regression analysis of the 2021 graduate cohort indicates that the intention to engage in voluntary activities post-graduation was higher among international graduates and among graduates who expressed stronger agreement with the importance of volunteering and with the influence of colleagues.

The analysis of the 2022 graduate cohort revealed a higher desire to engage in voluntary activities post-graduation among females, among graduates who expressed stronger agreement with the importance of volunteering, and the influence of other colleagues. Stronger agreement with the importance of improving the Curriculum Vitae showed a negative association with the interest in volunteering after graduation. 

The analysis of the 2023 graduate cohort indicated that the intention to engage in post-graduation voluntary activities was higher among females and among graduates who expressed stronger agreement with the importance of volunteering and helping the community.

The analysis of the Romanian graduate cohort indicates a higher intention to engage in voluntary activities post-graduation among females and among those who expressed stronger agreement with the importance of volunteering, graduates who perceived the opportunity to improve clinical knowledge and skills, and graduates who have a higher perception of the influence of other colleagues.

The analysis of the international graduate cohort indicates that intentions were higher among females and among graduates who expressed stronger agreement with the importance of volunteering and with intrinsic motivation and personal interest. (See Table 6).

**Table 6:** Multivariate linear regression - Factors associated with interest in volunteering after graduation

Cohort	Factors	Beta	CI	*P*
2021 Graduates	Section	0.249	(0.060–0.439)	< 0.05
	Importance of volunteering	0.285	(0.147–0.423)	< 0.001
	Influences from other colleagues	0.099	(0.008–0.191)	< 0.05
2022 Graduates	Importance of volunteering	0.280	(0.129–0.430)	< 0.001
	Gender	0.381	(0.150–0.611)	< 0.01
	Influences from other colleagues	0.194	(0.077–0.311)	< 0.01
	Desire for improving CV	-0.172	(-0.276- -0.068)	< 0.01
2023 Graduates	Importance of volunteering	0.439	(0.278–0.599)	< 0.001
	Gender	0.388	(0.167–0.609)	< 0.01
	The desire to help the community	0.146	(0.020–0.272)	< 0.05
Romanian Graduates	Importance of volunteering	0.332	(0.231–0.432)	< 0.001
	Gender	0.156	(0.011-0.300)	< 0.05
	Influences from other colleagues	0.079	(0.011–0.147)	< 0.05
	Desire to improve clinical knowledge and skills	0.071	(0.001–0.140)	< 0.05
International graduates	Importance of volunteering	0.307	(0.131–0.482)	< 0.01
	Gender	0.386	(0.152–0.621)	< 0.01
	Motivation and personal interest	0.267	(0.039–0.495)	< 0.05

Legend: Statistical significance was considered at *p* < 0.05

## Discussion

This study assessed volunteering among medical graduates during their studies in Romania during a three-year period (2021–2023). At the European level, a study performed in 2010 highlighted major differences in volunteering across countries: while Ireland, the Netherlands, and the UK have strong and well-developed sectors, countries such as Bulgaria, Greece, Latvia, Lithuania, and Romania have less developed ones, reflecting historical, political, and cultural contexts [17].

Our study shows that at Iuliu Hatieganu University of Medicine and Pharmacy from Cluj-Napoca, Romania, most medical graduates acknowledged the importance of volunteering.

Approximately 94% of Romanian and 92% of international graduates rated volunteering as important or very important. No significant differences were detected between the two sections or the three graduate cohorts. More than 80% of Romanian graduates and 83% of international graduates strongly agreed or agreed that several personal factors played an important role in making students get involved.

Based on the mean score for Romanian graduates, the most important personal factors were motivation and personal interest, followed by the desire to improve clinical knowledge and skills and the desire to gain experience in clinical reasoning and case management. A similar situation was found among international graduates. At the same time, we noticed statistically significant differences between the two sections, international graduates were more convinced about the role played by the desire to gain experience in clinical reasoning and case management.

Approximately 90% of Romanian and international graduates strongly agreed or agreed that several community factors played an important role in making students get involved. The highest-rated factors were opportunities for socialization and interpersonal relationships, and the desire to help the community. Statistically significant differences were observed between the two groups, with international graduates placing greater emphasis on the importance of helping the community.

More than 87% of Romanian graduates and 84% of international graduates strongly agreed or agreed that the most important educational factor was the desire to contribute to medical education of different population groups. A statistically significant difference existed between the cohorts of 2021 and 2022 graduates, with 2021 graduates showing a greater influence of the previously mentioned educational factors.

Looking globally to all three types of motivational factors among Romanian the three most important factors in influencing involvement in volunteer was considered to be motivation and personal interest, followed by opportunities for socialization and interpersonal relationships and the desire to contribute to medical education of different population groups. Among international graduates, the three most significant factors influencing engagement in volunteer activities were motivation and personal interest, followed by the desire to help the community and to contribute to medical education of different population groups. Comparing the three cohorts, the three most significant factors influencing involvement in volunteer activities were similar to those observed in the Romanian section.

To sustain our outcome, a prior study conducted in 2021 among IHUMP students in medicine, dentistry, nursing, and pharmacy showed that participants recognized the need to support pandemic control, were motivated to help others and public health authorities, and sought to develop transferable skills to strengthen their medical abilities and CVs [18]. Two similar studies conducted regarding the volunteering during the pandemic in Poland and in Bulgaria reflect a balance between altruism and professional development. In Poland, the dominant drivers were moral duty and solidarity with healthcare workers, students reporting high personal satisfaction, while in Bulgaria, most students viewed volunteering as an opportunity to gain experience and strengthen their professional identity while also fulfilling a social responsibility [19, 20].

Research in Ireland and the UK highlighted comparable patterns. A study among medical students from National University of Ireland, Galway and focused on attitudes of medical students toward volunteering in emergency situations identified altruism, skill acquisition, training in emergency response, and CV enhancement as major motivators, with concern for others ranked highest [3]. A former investigation among students from Imperial College School of Medicine UK, showed that 98% of students cited altruism as their primary reason, while 28% of respondents co-expressed additional self-directed motives for volunteering, such a need to learn or to avoid boredom [21].

Volunteering practices differ worldwide, shaped by cultural, social, and educational influences. Prior studies categorized student volunteers into three types: Independent (self-organized), facilitated (mediated by institutions or student organizations), and academic (assessed or monitored voluntary activities related to their education, such as service learning) [14]. Volunteer work encompasses a variety of activities, humanitarian help, blood donation, mentorship programs, skill-based contributions, and participation in emergency and medical support services.

In Romania, volunteering is an extracurricular activity, and students could be enrolled in various voluntary activities from their first year until graduation as independent student volunteers or as facilitated student volunteers, being mediated by student organizations. The IHUMP, Cluj-Napoca, has an affiliated student organization- Medical Students’ Organization from Cluj (MSOC), which offers many opportunities for students to volunteer. Substantial assistance was demonstrated by student volunteers during the COVID-19 pandemic. They assisted Public Health Directorates, which were essential in preventing new infections through case investigations and contact tracing from the onset of the pandemic. Additionally, student volunteers participated in healthcare settings by collecting patients’ medical histories, aiding healthcare professionals, and delivering direct care or performing minor procedures for patients. Volunteers received benefits based on their volunteer hours: 30 h for active status in the local student organization, and 120 h for mandatory clinical clerkship credits. This was an important move in recognizing the benefits of student volunteerism [18].

Our findings confirm that Romanian graduates remain active volunteers, mainly within Medical Students’ Organization from Cluj (MSOC), where opportunities are accessible through peer networks.

Romanian graduates participated at a rate of over 80%, compared to international graduates, who were engaged at a rate below 50%. Nevertheless, half of the Romanian graduates expressed interest in participating in voluntary activities after graduation, compared to 67% of the international graduates, indicating a statistically significant higher interest among the international graduates. This difference, consistently observed across all three cohorts, may be partly explained by the presence of language or cultural barriers that limited international graduates’ involvement during their studies in Romania. However, once they return to their home countries, international graduates may find volunteering opportunities more accessible or more strongly encouraged by their local healthcare systems.

The results of multivariate regression show that involvement in volunteering activities was higher among Romanian nationals for all three cohort groups, but belonging to the international section was one of the most important factors associated with intention to get involved after graduation only for 2021 graduates. Girls were more involved in research activities among 2023 graduates, and girls declared a higher intention to get involved after graduation for both sections and for all cohorts, except 2021.

Simultaneously, valuing the importance of volunteering was one of the most important factors positively associated with involvement and intention to get involved for both sections and all cohorts, except for 2023 graduates.

Involvement or intention to get involved were associated with strongest attitudes about the role of different factors. Among Romanian graduates, involvement in volunteering was positively associated with attitudes in favor of influences from other colleagues, and younger age. Among international graduates, stronger recognition of motivation and personal interest were positively associated with volunteering involvement. Among international graduates stronger agreement with the role of volunteering for gaining experience in clinical reasoning and case management was negatively associated with volunteering involvement, indicating that graduates most involved in volunteering were less convinced that volunteering contributes to the development of clinical reasoning and case management skills, this is probably in connection with the type of volunteering activities they performed. 

 Regarding intentions, these were influenced mainly by paying more attention to influences from other colleagues and the desire to improve clinical knowledge and skills among Romanian graduates, while among international graduates, intentions were higher among those who expressed stronger agreement with the importance of motivation and personal interest.

On the other hand, among 2021 graduates, volunteering was more frequent among those who placed higher importance on motivation and personal interest, while among 2022 graduates, it was positively associated with stronger beliefs about the opportunity for socialization and interpersonal relationships. Also, among the 2022 cohort, stronger agreement with the role of volunteering for gaining experience in clinical reasoning and case management was negatively associated with volunteering involvement. 

The intention to get involved in volunteer activities was higher among those who reported stronger influence from colleagues in both 2021 and 2022 cohorts, while among 2023 graduates the intention was more strongly linked to stronger agreement about the importance of helping the community. Among 2022 graduates, a negative association was observed between stronger agreement with the desire to improve the curriculum vitae and the intention to engage in post-graduation volunteering. This may indicate that students who attached greater importance to this factor during medical school no longer considered additional volunteering necessary after graduation.

Looking deeper to the type of volunteering activities, we noticed that popular activities included student exchanges, organizational work, health education, and medical support, while participation in emergency services was less common, likely due to the requirement of completing a course and passing an examination for enrollment; consequently, engagement may be more difficult, and constraints of time and current schedules could affect the decision to participate in such activities. Students must dedicate time to attending classes before enrollment, and the program may subsequently be exhausting since they predominantly engage in on-call work.

In the United States, medical students can participate in various volunteer options, including working in free clinics, aiding marginalized populations, delivering health education, and supporting public health programs. A few medical schools have integrated Service-Learning into their curriculum, which combines community service with educational planning and reflection. This approach helps academic student volunteers to understand their roles as health professionals and community members. Student-run clinics, integrated into service-learning curricula, provide supervised care to underserved populations and have expanded significantly in recent decades [22–24]. Despite these opportunities, many U.S. students do not engage in volunteering, and community service is not consistently embedded in medical school missions. However, the Association of American Medical Colleges found that only one-fifth of medical students who graduated said they had worked with various populations and only one-third said they performed field projects in service [22]. Unlike in other countries, Romania lacks student-run free clinics. Medical students instead participate in volunteer projects in which they provide free healthcare services under the direction of specialists and teachers. There are some projects in this field mentioned by our participants: ‘Vaccination Marathon’, ‘Doctors for You’, ‘Together for Health’, ‘Oncohelp’, ‘The Caravan with Doctors Association’.

A prior study described an interesting way to volunteer, aiming to improve geriatric assessment abilities, promote good attitudes toward senior patients, and raise geriatrics knowledge. The Uniformed Services University developed a new geriatrics home visit program. While all participants learned about geriatrics during their internal medicine clerkship, students who performed home visits had better views of the elderly and described using geriatric assessment abilities [25]. A recent study among Penn State College of Medicine students regarding volunteerism through blood donation revealed that most would be ready to donate blood amid a mass casualty event [26]. Similarly, our findings showed high interest in participating in the campaign ‘Donate Blood! Be a Hero!’ Project, developed by MSOC. Present in numerous colleges and medical institutions worldwide and in Romania, Teddy Bear Hospital is a student-led voluntary association that uses play to encourage good living among kids in primary school (aged 5 to 7 years) and help them to lower their anxiety about hospitals and doctors [27].

This study has several limitations. The primary limitation is that the findings may only be comparable to those from other medical schools that implement similar extracurricular voluntary initiatives. The research sample involves just medical graduates from a singular Romanian medical institution, so constraining the representation of varied viewpoints and experiences among the wider community of medical graduates. Furthermore, those with a greater interest in this domain may self-select, thereby affecting the results. A possible limitation of our study is the reported low response rate. Additionally, uncontrolled aspects such as financial background, experience, or detailed personal motives might distort the correlation between identified variables and volunteer engagement. Also, the cross-sectional design of the study may limit its capacity to detect variations in volunteer involvement. Observing trends and experiences throughout time or across several levels of medical education is extremely difficult.

## Conclusion

This study provides the first insight into volunteering behaviors and attitudes of Romanian medical graduates regarding their involvement in volunteering activities up until graduation. Although altruism and community responsibility remain determining attitudes, our findings show that professional development and networking opportunities are equally important. Furthermore, student organizations play a central role in facilitating opportunities to volunteer.

These findings suggest that incorporating structured voluntary activities into medical curricula, as well as leveraging efforts through credit recognition for service, may help maintain student involvement throughout medical school. Addressing cultural disparities is equally important to ensure equitable opportunities for all graduates to volunteer.

The findings carry practical implications for mentors, who can use them to design effective strategies for encouraging undergraduate participation in community service and to enhance cross-cultural education. Furthermore, they provide actionable insights for policymakers and medical institutions useful information that might help them create a collaborative framework that encourages volunteer involvement and its associated professional benefits.

Further investigation should be conducted to identify with stakeholders the optimal integration of insights gained from these activities into the formal curriculum, as well as to assess the medium- and long-term effects of volunteering on participants.

## Supplementary Information

Supplementary Material 1.

## Data Availability

The datasets utilized and analyzed in the present study are accessible upon reasonable request from the corresponding author.
