# Supplementary material for: Attitudes and practices regarding volunteering among medical graduates from Romania
Source: BMC Med Educ. 2026 Aug 20;26:1340. doi: 10.1186/s12909-026-09234-3 (PMC13491685; doi:10.1186/s12909-026-09234-3)
Supplement: Supplementary file 1 [file 12909_2026_9234_MOESM1_ESM.docx]

Supplementary File 1 – Survey Questionnaire

This section contains the survey questionnaire from which the data in the article was collected.

1.Gender

a)	Male

b)	Female

1. Year of graduation [Open-ended question]
2. Section [Open-ended question]
3. Age [Open-ended question]

5. What do you believe about medical students participating in voluntary activities?

a)	Very important

b)	Important

c)	Not at all important

d)	Undecided

6. Have you been involved in volunteer activities during your medical studies (Within the medical student organization)?

a)	Yes

b)	No

7. Have you been involved in volunteer activities during your medical studies (Within another organization)?

a)	Yes

b)	No

8. If the previous answers were yes, what voluntary activities did you take part in? [Open-ended question]

9. Do you want to be involved in volunteer activities after graduation?

a)	Yes

b)	No

c)	Undecided

10. In your opinion, what factors encourage students to participate in volunteer activities?

I.	The motivation and self-interest

a)	Strongly agree

b)	Agree

c)	Undecided

d)	Disagree

e)	Strongly disagree

II.	By showing the possibilities of participation in volunteer activities by teachers

a)	Strongly agree

b)	Agree

c)	Undecided

d)	Disagree

e)	Strongly disagree

III.	The knowledge and expertise of other colleagues who are active in a volunteer organization

a)	Strongly agree

b)	Agree

c)	Undecided

d)	Disagree

e)	Strongly disagree

IV.	Volunteer programs provide opportunities for socializing and interpersonal interaction.

a)	Strongly agree

b)	Agree

c)	Undecided

d)	Disagree

e)	Strongly disagree

V.	The desire to improve the cv

a)	Strongly agree

b)	Agree

c)	Undecided

d)	Disagree

e)	Strongly disagree

VI.	The desire to improve clinical knowledge and skills

a)	Strongly agree

b)	Agree

c)	Undecided

d)	Disagree

e)	Strongly disagree

VII.	The desire to gain experience in clinical reasoning and case management

a)	Strongly agree

b)	Agree

c)	Undecided

d)	Disagree

e)	Strongly disagree

VIII.	The desire to lend a helping hand to the community

a)	Strongly agree

b)	Agree

c)	Undecided

d)	Disagree

e)	Strongly disagree

IX.	The aim to contribute to the medical education of different population groups

a)	Strongly agree

b)	Agree

c)	Undecided

d)	Disagree

e)	Strongly disagree

X.	Others [Open-ended question]
